# Infratentorielle Hirntumoren bei Kindern

**DOI:** 10.1007/s00117-023-01159-y

**Published:** 2023-06-12

**Authors:** J. M Lieb, A. Lonak, A. Vogler, F. Pruefer, F. J. Ahlhelm

**Affiliations:** 1 Abteilung Neuroradiologie, Klinik für Radiologie und Nuklearmedizin, Departement Theragnostik, Universitätsspital Basel, Petersgraben 4, 4031 Basel, Schweiz; 2 Kinderradiologie, Universitäts-Kinderspital beider Basel, Basel, Schweiz; 3 Abteilung für Neuroradiologie, Zentrum für Bildgebung, Kantonsspital Baden AG, Baden, Schweiz

**Keywords:** Neoplasien des Zentralnervensystems, Medulloblastom, Pilozytisches Astrozytom, Ependymom, Magnetresonanztomographie, Central nervous system neoplasms, Medulloblastoma, Pilocytic astrocytoma, Ependymoma, Magnetic resonance imaging

## Abstract

**Klinisches Problem:**

Tumoren der hinteren Schädelgrube machen etwa 50–55 % der kindlichen Hirntumoren aus.

**Diagnostik:**

Zu den häufigsten Tumorentitäten zählen Medulloblastome, pilozytische Astrozytome, Ependymome, diffuse Mittelliniengliome und atypisch teratoid-rhabdoide Tumoren (ATRT). Der neuroradiologischen Differenzialdiagnostik mittels Magnetresonanztomographie (MRT) kommt eine erhebliche Bedeutung zu, sowohl für die präoperative Planung als auch für die Planung der Anschlusstherapie.

**Leistungsfähigkeit:**

Wichtige Merkmale für die Differenzialdiagnostik sind die genaue Tumorlokalisation, das Patientenalter und die intratumorale scheinbare Diffusion, die mittels diffusionsgewichteter Bildgebung quantifiziert werden kann.

**Bewertung:**

Fortschrittliche MR-Techniken, wie MR-Perfusion und MR-Spektroskopie, können sowohl für die initiale Diagnostik als auch für die Beurteilung des Tumorverlaufs hilfreich sein, allerdings sollten Ausnahmeverhalten bestimmter Tumorentitäten bekannt sein.

**Empfehlung für die Praxis:**

Konventionelle MRT-Sequenzen inklusive Diffusionswichtung sind die wichtigsten diagnostischen Tools zur Evaluation pädiatrischer Tumoren der hinteren Schädelgrube. Fortschrittliche MR-Techniken können helfen, sollten allerdings nicht isoliert von den konventionellen MRT-Sequenzen interpretiert werden.

## Abstract (en)

**Clinical issue:**

Tumors of the posterior fossa account for about 50–55% of brain tumors in childhood.

**Diagnostic workup:**

The most frequent tumor entities are medulloblastomas, pilocytic astrocytomas, ependymomas, diffuse midline gliomas and atypical teratoid-rhabdoid tumors. Neuroradiological differential diagnosis with magnetic resonance imaging (MRI) is of considerable importance for preoperative planning as well as planning of follow-up therapy.

**Performance:**

Most important findings for differential diagnosis of pediatric posterior fossa tumors are tumor location, patient age and the intratumoral apparent diffusion assessed by diffusion-weighted imaging.

**Achievements:**

Advanced MR techniques like MRI perfusion and MR spectroscopy can be helpful both in the initial differential diagnosis and in tumor surveillance, but exceptional characteristics of certain tumor entities should be kept in mind.

**Practical recommendations:**

Standard clinical MRI sequences including diffusion-weighted imaging are the main diagnostic tool in evaluating posterior fossa tumors in children. Advanced imaging methods can be helpful, but should never be interpreted separately from conventional MRI sequences.

Primäre Hirntumoren machen ca. 25 % aller Krebserkrankungen im Kindesalter aus und stellen die häufigsten soliden Tumoren bei Kindern und Jugendlichen dar; etwa 50–55 % der kindlichen Hirntumoren liegen infratentoriell [26].

Die histopathologischen sowie molekularen Charakteristika, Malignitätsgrade, Mestastasierungsfrequenz und Prognose variieren zwischen den verschiedenen Tumorentitäten. Der Neuroradiologie kommt in Bezug auf die Differenzialdiagnostik eine entscheidende Rolle zu, da eine akkurate Diagnose für das weitere Patientenmanagement und die Therapieplanung essenziell ist. Zu den häufigsten pädiatrischen infratentoriellen Hirntumoren zählen in absteigender Reihenfolge das Medulloblastom, das pilozytische Astrozytom (PCA), das Ependymom, das diffuse Mittelliniengliom und der atypische teratoid-rhabdoide Tumor (ATRT).

## Medulloblastome

Medulloblastome sind High-grade-Tumoren Grad 4 nach der Klassifikation der Weltgesundheitsorganisation (WHO) und mit 25–30 % die häufigsten pädiatrischen Hirntumoren. Gleichzeitig stellen sie auch die häufigsten malignen primären Tumoren der hinteren Schädelgrube beim Kind dar [14, 22].

Gemäß der neuesten WHO-Klassifikation von 2021 werden für die Typisierung der Medulloblastome deren molekulare Profile berücksichtigt. Diese sind abhängig von charakteristischen Mutationen/Genamplifikationen, z. B. über die Aktivierung der Signalwege „sonic hedgehog“ (SHH) oder „wingless/integrated“ (WNT).

Medulloblastome der Gruppe 3 und 4 (Non-WNT/Non-SHH, histologisch meist klassisch) sind Mittellinientumoren, jedoch neigen manche Subtypen zu einer Off-midline-Manifestation. Allen gemeinsam ist die Eigenschaft dicht gepackter Zellen. In der Bildgebung korreliert der Zellreichtum und die Zelldichte der Medulloblastome in der Computertomographie (CT) mit entsprechender Hyperdensität und in der Magnetresonanztomographie (MRT) mit einer Diffusionsrestriktion.

In der Regel sind Medulloblastome rundlich oder ovalär konfiguriert, zeigen hyper- und hypointense Areale in T2w verglichen zum Kleinhirnparenchym und nehmen fleckig bis homogen Kontrastmittel auf [10, 23, 27]. Medulloblastome können metastasieren, typischerweise über eine Liquorraum-Aussaat und in Abhängigkeit vom molekularen Typ/Subtyp. Die Metastasierungsrate bei Diagnosestellung liegt zwischen 10 % beim WNT-Subtyp und bis zu 45 % bei den Gruppe-3-Medulloblastomen, daher gehört die Abklärung der spinalen Achse zum präoperativen Standard und auch zur Tumornachsorge [22].

Im Folgenden gehen wir auf die verschiedenen Typen von Medulloblastomen ein (Tab. 1).

**Table 1:** 

WHO 2021 (Typen)	MB, WNT-aktiviert	MB, SHH-aktiviert, TP53-Wildtyp	MB, SHH-aktiviert, TP53-mutiert	MB, Non-WNT/Non-SHH	
WHO 2016 (molekulare Subtypen)	WNT	SHH		Gruppe 3	Gruppe 4
Häufigste anatomische Lokalisation	Dorsaler Hirnstamm	Cerebelläre Hemisphären		Mittellinie	Mittellinie
Häufigkeit innerhalb der Medulloblastome	10 %	30 %		25 %	35 %
Angenommener Zellursprung	Vorläuferzellen der unteren rhombischen Lippe	Vorläuferzellen der externen Granularzellen		Vorläuferzellen der oberen rhombischen Lippe	
5‑Jahres-Überlebensrate	95–100 %	80 %	40 %	Ca. 30–60 %	75 %
Betroffene Altersgruppen	Kinder und Erwachsene	Kinder	Kleinkinder und Erwachsene (bimodal)	Kinder	Kinder
Histologie	Klassisch	Klassisch, desmoplastisch, großzellig/anaplastisch		Klassisch, desmoplastisch, großzellig/anaplastisch	Klassisch
Metastasierungshäufigkeit (je nach Subtyp)	12 %	20 %	9–30 %	35–62 %	45–50 %
Geschlechtsverteilung (je nach Subtyp)	♂ 45 % ♀ 55 %	♂ 47–63 % ♀ 31–53 %		♂ 60–71 % ♀ 29–40 %	♂ 66–75 % ♀ 25–33 %

*SHH* „sonic hedgehog“, *WNT* „wingless type“

WNT-aktivierte Medulloblastome sind der seltenste Medulloblastomtyp (ca. 12 %) mit der besten Prognose. Meist sind ältere Kinder oder junge Erwachsene betroffen. Sie entstehen typischerweise an der unteren rhombischen Lippe. WNT-aktivierte Medulloblastome können in der Mittellinie, aber auch „off-midline“ in der Nähe der Foramina Luschkae, der Kleinhirnstiele oder im Kleinhirnbrückenwinkel auftreten [8, 10, 24].

**Figure 1:**
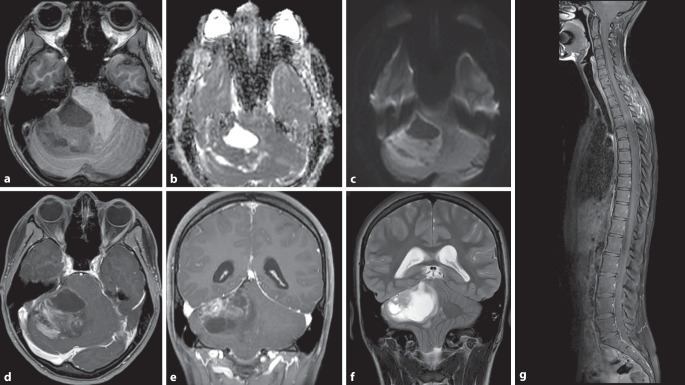

SHH-aktivierte Medulloblastome (Abb. 1) sind eine heterogene Gruppe, machen etwa 30 % der Medulloblastome aus und zeigen eine mittlere Prognose. Sie gehen wahrscheinlich aus Vorläuferzellen der externen Granularzellschicht hervor, die ihren Ursprung an der oberen rhombischen Lippe haben [17]. Diese Medulloblastome treten eher lateral in den Kleinhirnhemisphären auf (Abb. 1), können aber auch in der Mittellinie vorkommen [8, 12]. Die Altersverteilung ist bimodal, meist sind Säuglinge oder Kleinkinder betroffen, dann junge Erwachsene und selten Kinder. Die Kleinkindvariante metastasiert häufiger [7, 12].

Non-WNT/Non-SSH-aktivierte Medulloblastome (Gruppe 3 und Gruppe 4) stellen die häufigste molekulare Subgruppe der Medulloblastome dar. Beide Typen entspringen wahrscheinlich einer gemeinsamen Zelllinie in der oberen rhombischen Lippe [27] und sind primär Mittellinientumoren. Sie treten häufiger bei Jungen auf als bei Mädchen und können klassische, desmoplastische oder anaplastische histologische Merkmale aufweisen.

**Figure 2:**
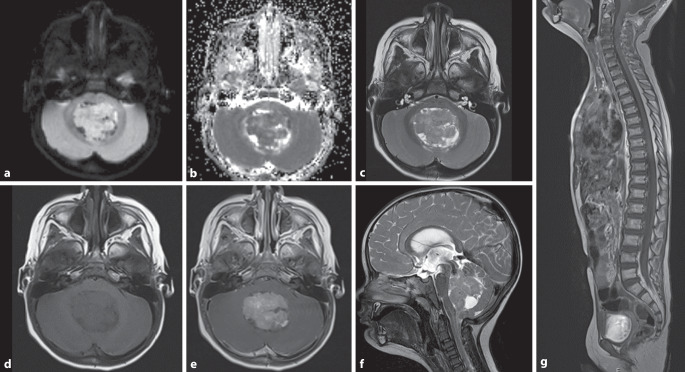

**Figure 3:**
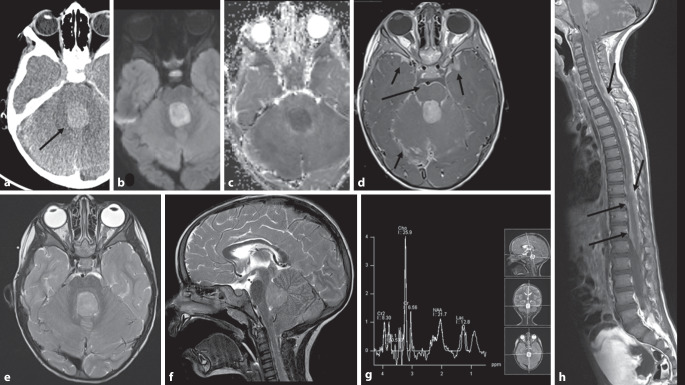

Die Gruppe-3-Medulloblastome (Abb. 2 und 3, etwa 25 %) haben die schlechteste Gesamtprognose, metastasieren häufiger (Abb. 3) und kommen in der Regel bei (Klein‑)Kindern vor.

Gruppe-4-Medulloblastome gehören mit etwa 35 % zum häufigsten Typ. Sie kommen bei älteren Kindern und jungen Erwachsenen vor und zeigen eine intermediäre Prognose [8, 12]. Bildgebend können sich beide Gruppen durch die Kontrastmittelaufnahme unterscheiden: Während Medulloblastome der Gruppe 4 eher wenig Kontrastmittel aufnehmen, zeigen Gruppe-3-Tumoren häufig ein sehr starkes KM-Enhancement [25].

### Pilozytische Astrozytome

**Figure 4:**
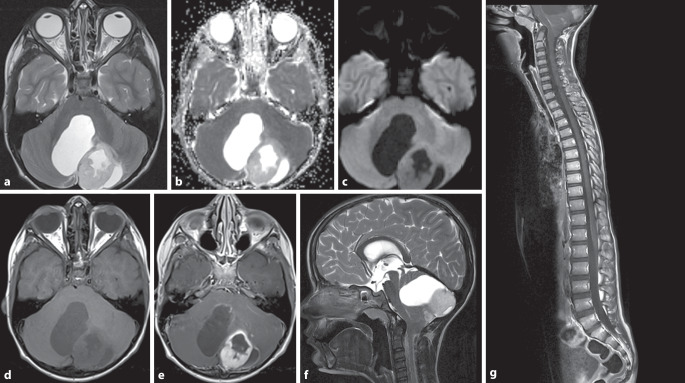

Beim PCA (Abb. 4), einem WHO-1-Tumor, handelt es sich um den zweithäufigsten kindlichen Hirntumor der hinteren Schädelgrube. Im Fall einer vollständigen Resektion hat das PCA eine exzellente Prognose. Es entsteht durch Änderungen im MAPK-Pathway und zeigt oft BRAF-Fusionen oder BRAF-V600E-Punktmutationen. Patienten mit BRAF-Fusion zeigen ein etwas besseres Outcome [5]. Sie treten gehäuft bei Patienten mit Neurofibromatose 1 (NF1) auf, und dort am häufigsten im Verlauf der Sehbahn [2]. Meist entstehen pilozytische Astrozytome in den Kleinhirnhemisphären und sind lateral lokalisiert. Seltener gehen sie mittig vom Kleinhirnwurm aus. Bildgebend klassisch ist eine zystische Raumforderung mit einem randständigen, soliden Knoten (Abb. 4). Heterogen-multizystische oder mehr solide Formen mit zentral-zystischen Veränderungen sind seltener, ebenso auch hämorrhagische Varianten [10, 21]. Die zystische Komponente zeigt in der MRT häufig ein liquorisointenses Signal in T1w und T2w, während das FLAIR-Signal (Fluid-Attenuated Inversion Recovery) im zystischen Anteil je nach proteinreichem Inhalt variieren kann. Die soliden Anteile dieser Tumoren nehmen typischerweise sehr kräftig Kontrastmittel auf, zeigen aber keine Diffusionsrestriktion (Abb. 4; [13, 20]). Die Zystenwand kann ebenfalls Kontrastmittel aufnehmen.

### Ependymome

**Figure 5:**
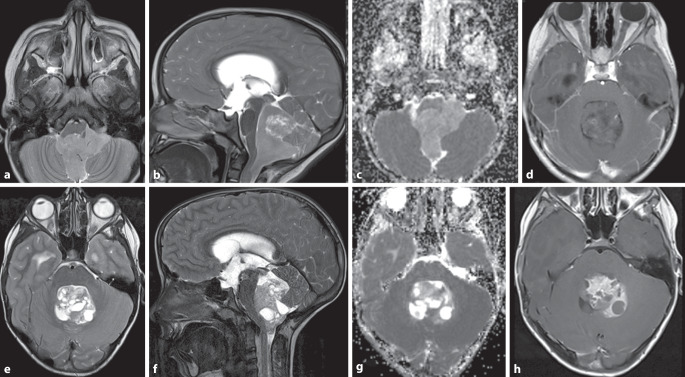

Ependymome sind die dritthäufigsten infratentoriellen Hirntumoren bei Kindern. In der Regel handelt es sich um klassische WHO-Grad-2-Tumoren, es kann aber auch ein Grad 3 mit aggressiveren anaplastischen Merkmalen vorliegen. Typisch ist ihr plastisches Wachstum entlang vorgegebener Strukturen. Meist entstehen sie im oder in der Nähe des 4. Ventrikels und wachsen durch die Foramina Luschkae in die benachbarten Zisternen. Es existieren 2 Typen der Ependymome in der hinteren Schädelgrube, Gruppe A und B (Abb. 5; [17]).

Gruppe-A-Ependymome treten häufiger bei Kleinkindern auf und mehr lateral mit Ausdehnung nach präpontin, was eine vollständige Resektion erschwert. Gruppe-B-Ependymome treten bei älteren Kindern und jungen Erwachsenen auf, gehen meist vom 4. Ventrikel aus, sind eher mittig lokalisiert und haben eine insgesamt bessere Prognose [2, 31].

Bildgebend zeigen Ependymome häufig ein heterogenes hyperintenses Signal in T2w mit variabler Kontrastmittelaufnahme. Deutlich häufiger als Medulloblastome zeigen etwa 50 % der Fälle zystische Veränderungen oder Verkalkungen (Abb. 5e–h; [10]). Aufgrund ihres neuroplastischen Wachstums und der weichen Konsistenz ist die Ausdehnung über die Foramina Luschkae ein charakteristisches Merkmal (Abb. 5a–c). Ependymome können eine Diffusionsrestriktion zeigen, typischerweise aber weniger ausgeprägt als Medulloblastome [32].

Die Metastasierungsrate über den Liquorraum liegt für Ependymome WHO 3 höher als für Ependymome WHO 2, letztere liegen hinsichtlich Frequenz aber unterhalb der Metastasierungsrate von Medulloblastomen [32]. Die Abklärung der spinalen Achse gehört also auch hier zum präoperativen Standard und zur Nachsorge.

### Diffuses Mittelliniengliom

Diffuse Mittelliniengliome (DMG, vormals diffuse intrinsische Ponsgliome) mit Histon-H3K27-Alteration sind hochaggressive WHO-Grad-4-Tumoren und haben mit einem medianen Überleben von ca. 11 Monaten ab Diagnosestellung eine schlechte Prognose [9]. Das mittlere Alter bei Diagnosestellung liegt etwa bei 6 Jahren [15].

Die häufigsten klinischen Symptome sind Hirnnervenlähmungen, Pyramidenbahnzeichen und cerebelläre Symptome [9]. Aufgrund der sehr häufigen Hirnstammlokalisation fehlen chirurgische Optionen.

**Figure 6:**
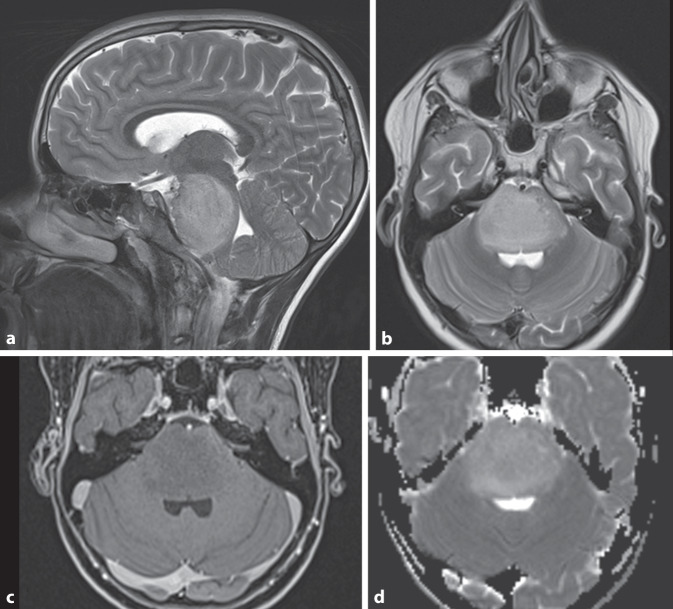

Bildgebend handelt es sich um diffuse, unscharf berandete, in T2W und FLAIR-Technik hyperintense raumfordernde Signalalterationen des Pons (Abb. 6), häufig anfänglich ohne Kontrastmittelaufnahme und im Verlauf – wenn auftretend – eher fleckig.

Nach Radiotherapie treten häufig nekrotische Veränderungen mit randständigem KM-Enhancement auf [1]. Diese Veränderungen mit KM-Rand-Enhancement und Diffusionsrestriktion initial bei Diagnosestellung gelten als schlechte prognostische Zeichen [15]. Lange Zeit wurden diffuse Mittelliniengliome des Hirnstamms bei charakteristischen bildgebenden Befunden ohne histologische Sicherung behandelt. Zwischenzeitlich werden häufiger Biopsien zur Untersuchung des molekularen Tumorprofils durchgeführt, um potenzielle Therapietargets zu evaluieren [6].

### Atypischer teratoid-rhabdoider Tumor

Bei den ATRT handelt es sich um seltene, hochaggressive WHO-Grad-4-Tumoren embryonalen Ursprungs. Die meisten dieser zellreichen Tumoren treten bei Kindern < 3 Jahren auf. Ihre Lokalisation in der hinteren Schädelgrube kann, ähnlich wie beim Medulloblastom, mittig oder „off-midline“ sein.

Die MRT-Charakteristika von ATRT überschneiden sich deutlich mit denen der Medulloblastome, daher ist das sehr junge Erkrankungsalter einer der Schlüssel zur Diagnose. ATRT sind häufig etwas inhomogener, bspw. zeigen sie häufiger intratumorale Einblutungen oder Verkalkungen als Medulloblastome. Oft liegt zum Diagnosezeitpunkt bereits eine Metastasierung über den Liquorraum vor [4, 11]. Eindeutige bildgebende Unterscheidungsmerkmale zwischen Medulloblastomen und ATRT existieren nicht.

Neben den 5 häufigsten, bereits beschriebenen pädiatrischen Hirntumoren in der hinteren Schädelgrube gibt es dort auch weitere Tumorarten, die seltener vorkommen. Hierzu zählen diffuse Astrozytome, High-grade-Astrozytome, Glioblastome und auch Metastasen.

## Fortschrittliche MRT-Techniken

### Diffusionsgewichtete Bildgebung

Aufgrund der hohen Zellularität des Medulloblastoms spielt die Diffusionswichtung (DWI) eine große Rolle bei der bildgebenden Differenzialdiagnostik. So konnte in einem Kollektiv bspw. mittels Quantifizierung der scheinbaren Diffusion über sog. Apparent-diffusion-coefficient(ADC)-Parameterkarten ein Medulloblastom mit einer Sensitivität und Spezifität von 95,8 % und 81 % diagnostiziert werden [29].

Eine große Metaanalyse zur diagnostischen Effizienz und Accuracy der DWI bei der Differenzierung zwischen Medulloblastomen und anderen Tumorentitäten in der hinteren Schädelgrube zeigte sogar eine gepoolte Sensitivität/Spezifität von 0,95/0,94 und eine *exzellente* Accuracy [18].

**Figure 7:**
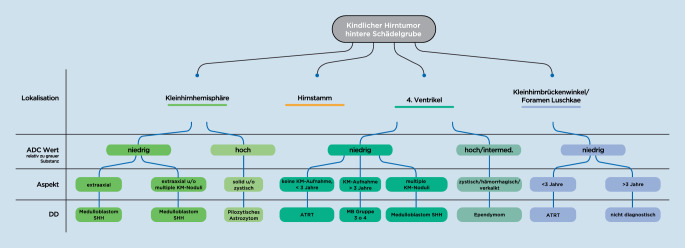

Alves et al. entwickelten ein Flowchart zur Differenzialdiagnostik von kindlichen Hirntumoren in der hinteren Schädelgrube, das neben der Lokalisation die scheinbare Diffusion berücksichtigt [3]. In Abb. 7 ist dieses Flowchart in adaptierter Form dargestellt.

### MR-Perfusion

MR-Perfusionstechniken können die Tumorvaskularität als auch Hämodynamik evaluieren. Eine Perfusionsuntersuchung in der MRT kann entweder kontrastmittelgestützt oder kontrastmittelfrei erfolgen. Kontrastmittelgestützt unterscheidet man zwischen T2*w-basierter („dynamic susceptibility contrast“, DSC) und T1w-basierter („dynamic contrast enhanced“, DCE) MR-Perfusion. Kontrastmittelfreie Perfusionen basieren bspw. auf ASL („arterial spin labeling“).

Je nach Methode können unterschiedliche hämodynamische Parameter, wie z. B. relativer Blutfluss (rCBF), relatives Blutvolumen (rCBV), „time to peak“ (TTP), „mean transit time“ (MTT), vaskuläre Permeabilität oder der Transfer-Koeffizient (K-trans) berechnet werden. Alle diese Parameter können einen Einblick in die Tumorbiologie geben, wie bspw. den Malignitätsgrad. Sie können aber im Krankheitsverlauf auch bei der Differenzierung von Therapieansprechen, Rezidiv oder Therapiefolgen helfen [16].

Ein hohes für Leakage korrigiertes relatives zerebrales Blutvolumen („corrected rCBV“) korreliert häufig mit einem höheren Malignitätsgrad bei Hirntumoren, jedoch gibt es Tumorentitäten, die bezüglich ihres Blutvolumens höher- oder niedriggradige Tumoren imitieren können. Beispiele hierfür sind das Medulloblastom und das PCA. Während das Medulloblastom als aggressiver WHO-Grad-4-Tumor nicht zwingend ein hohes Blutvolumen zeigt, findet sich ein solches aber häufig beim niedriggradigen PCA [16]. Generell sollten MR-Perfusionsparameter daher nicht ohne die konventionellen MR-Charakteristika interpretiert werden.

### MR-Spektroskopie

Zusätzlich zur Information über Zellularität und Tumorvaskularität erlaubt die MR-Spektroskopie (MRS) Aussagen über die biochemische Zusammensetzung des untersuchten Gewebes. Mittels Proton-MR-Spektroskopie können viele verschiedene Metabolite abhängig von der verwendeten Echozeit („time of echo“, TE) bestimmt werden.

Die MRS kann in der primären Differenzialdiagnostik und auch bei Tumorverlaufskontrollen helfen, um Rezidive von therapieassoziierten Veränderungen abzugrenzen. Ein typisches Tumorspektrum zeigt eine Erhöhung von Cholin und eine Erniedrigung von NAA (Abb. 3g). Je höhergradiger ein Tumor ist, desto ausgeprägter sind in der Regel diese spektralen Veränderungen. Hochgradige maligne, zellreiche Tumoren mit schnellem Wachstum zeigen hohe Cholin-Level, eine starke NAA-Erniedrigung sowie Laktat-Peaks, wenn das Tumorwachstum die Neoangiogenese überholt und sich eine anaerobe Glykolyse einstellt. Lipid-Peaks lassen sich in nekrotischen oder auch zystischen Arealen nachweisen.

Allerdings sollte eine MR-Spektroskopie nie ohne die konventionellen MRT-Sequenzen beurteilt werden, da es durchaus Tumorentitäten gibt, die spektral High-grade-Tumoren imitieren können, wie z. B. das PCA [16].

## WHO-Klassifikation 2021 – Fokus kindliche Hirntumoren

Die WHO-CNS-5-Klassifikation von 2021 löst die revidierte WHO-CNS-4-Klassifikation von 2016 ab mit zahlreichen Neuerungen in Bezug auf die pädiatrischen ZNS-Neoplasien. Es kamen nicht nur zahlreiche Tumorentitäten hinzu, wurden umbenannt oder gelöscht, sondern es wurde auch der immer zentraler werdenden molekularen Typisierung Rechnung getragen [30].

Zusätzlich wurde die Nummerierung des Tumorgrades von römischen Zahlen auf arabische Zahlen umgestellt [17, 19, 30]. Je nach Tumortyp können Histologie, molekulare Charakteristika und zum Teil auch anatomische Lokalisation in unterschiedlicher Weise diagnostische, prognostische und/oder prädiktive Aussagen erlauben. Daher ist der zentrale Ansatz der WHO-ZNS-5-Klassifikation eine geschichtete integrierte Diagnosestellung, die folgende sich komplementierende Faktoren beinhaltet:

- histologischer Grad,
- molekulare Typisierung,
- anatomische Lokalisation (in einzelnen Fällen),
- Tumorgrad, der die Aggressivität der Neoplasie in ihrem nicht therapierten Verlauf widerspiegelt.

Hierbei ist zu berücksichtigen, dass der Tumorgrad in der WHO-CNS-5-Klassifikation eine Gradierung der Aggressivität einer Neoplasie innerhalb derselben Entität abbildet und nicht – wie in der vorherigen Klassifikation von 2016 – eine Vergleichbarkeit einer Tumoraggressivität zwischen verschiedenen Entitäten anstrebt [19, 30].

Erstmalig wurde zudem berücksichtigt, dass zahlreiche kindliche Neoplasien – selbst wenn sie histologisch identisch zu Neoplasien bei Erwachsenen sind – eine andere Tumorbiologie aufweisen [19, 30].

Zusammenfassend sind in Bezug auf kindliche Hirntumoren und ihre Einordnung in die neue WHO-CNS-5-Klassifikation von 2021 folgende zentrale Kernpunkte hervorzuheben: Erstens wird die altersspezifisch deutlich unterschiedliche Tumorbiologie von Neoplasien des zentralen Nervensystems bei Kindern bzw. Erwachsenen neu betont, indem erstmals pädiatrische diffuse High-grade-Gliome als eigene Kategorie getrennt von High-grade-Gliomen im Erwachsenenalter aufgeführt werden. Zweitens bekommt die molekulare Signatur einen größeren Stellenwert. Im Falle der pädiatrischen Hirntumoren der hinteren Schädelgrube wirkt sich dies besonders stark auf die Medulloblastome aus. So wurden bspw. neue Medulloblastomtypen ergänzt in Abhängigkeit der SHH/WNT-Aktivierung und TP53-Mutation (Tab. 1).

## Fazit für die Praxis

- Tumore der hinteren Schädelgrube machen mehr als die Hälfte aller kindlichen Hirntumoren aus.
- Die häufigsten Tumorentitäten sind das Medulloblastom, das pilozytische Astrozytom (PCA), das Ependymom, das diffuse Mittelliniengliom und der atypisch teratoid-rhabdoide Tumor (ATRT).
- Sowohl Erkrankungsalter, Malignitätsgrad, Metastasierungstendenz als auch Prognosen der Entitäten variieren erheblich.
- Bildgebend charakteristisch für das klassische Medulloblastom (WHO Grad 4) ist ein Mittellinientumor mit hoher Zelldichte und entsprechend Diffusionsrestriktion.
- Charakteristisch für das pilozytische Astrozytom (WHO Grad 1) ist ein zystischer Tumor mit randständig kontrastmittelaffinem Knoten ohne Diffusionsrestriktion.
- Charakteristisch für das Ependymom ist das plastische Wachstum entlang vorgegebener Strukturen, während sich diffuse Mittelliniengliome meist als diffuse, raumfordernde Signalalteration des Pons manifestieren.
- ATRT zeigen deutliche bildgebende Überlappung mit dem Medulloblastom, treten jedoch i. d. R. bei Kindern < 3 Jahre auf.
- Mit Ausnahme des PCA neigen alle Tumoren zur Metastasierung über eine Liquorraum-Aussaat, weshalb die Magnetresonanztomographie (MRT) der spinalen Achse zum Tumorstaging und -überwachung gehört.
- Sowohl Alter, Tumorlokalisation als auch die Diffusionswichtung spielen eine zentrale Rolle bei der Differenzialdiagnostik.
